# Case of Extra Uterine Pregnancy—Death

**Published:** 1879-01

**Authors:** Richard A. Kennedy


### CASE OF EXTRA UTERINE PREGNANCY—DEATH.

BY RICHARD A. KENNEDY, M. D., C. M.

Mrs. A.—I first saw her in the beginning of February last, suffering from what I was led to believe, a threatened abortion, She considered herself to be pregnant with her second child. There was a bloody discharge per vaginum, great pain in the pelvis, vomiting and high fever, with great tenderness of the abdomen, which I diagnosed to be a localized peritonitis. She was six days under my treatment, and then went to the Hotel Dieu, under Dr. Hingston. She came out of the Hotel Dieu after a short term.

On the 24th Febuary, I again saw her, but do not remember the circumstances of my attendance, though she stated I gave her something which relieved her. I did not see her again until the 24th July, when she called at my office to pay something on her account. At that time she called my attention to her condition. The abdominal enlargement being that of a woman at about the 6th month of pregnancy; she complained of the fœtal movements, and at her request I placed my hand on her abdomen and am positive that I distinctly felt them. Of course not expecting but what it was an ordinary case of pregnancy, and that as usual it was all right, I did not examine her as closely as I now wish I had done. Her calculation was that confinement would take place about the middle of October, for which she wished to engage me. Early in August she called and stated that she feared the child was dead; she had hurt herself getting out of bed and had felt no movement since. The abdomen I found was larger than at the previous time when my attention was called to it. There was no fœtal movement, nor could I detect fœtal pulsation; as there was no indication of uterine action, I counseled her to keep quiet and wait. At a subsequent examination I thought I could detect the placental souffle, which was faint, and I thought that probably some circulation was continued in the fœtus, which might account for there being no attempt at labor. From the end of August she began to run down in health, got remarkably thin and debilitated, and had the appearance of a person suffering from the *absorption of septic matter*, chills and feverishness. I had considered the advisability of inducing labor; this she was averse to, and so were her friends, so that I placed her on iron and quinine, with a good diet.

About the beginning of September she complained of great pain in the right *inguinal region.* It was extremely tender to the touch, and there was an enlarged and distinct bulging; my opinion at the time was that the fœtus was dead and had changed its position, the body getting into a transverse direction with the head in the right side; this was apparently confirmed by the altered shape of the uterine tumor. At this time I did not consider it advisable, even if allowed, to induce labor, and by making her lie on this side with a pillow under the swelling (which could be pushed downwards), it disappeared, leading me to believe that the body had again assumed its usual position. I wanted again to induce labor, but she preferred to wait, as she thought the child might be alive and labor would come on in due time.

She suffered severely from pain and diarrhœa with fetid dejections, and had a bad cough; morphine was given for the relief of pain, also a cough mixture, and the quinine continued. By the end of September she continued to improve, got stronger, but she was also getting smaller, and on percussion there was evidence of gas or air in the tumor where it was before quite dull as in pregnancy.

At her own request I did not interfere as she considered her time to be up in the middle of October. The opinion that I now formed was that the child being dead had decomposed with the formation of gases and absorption of putrefactive matter which had been going on for some time. During the second week of October she sent for me, believing herself to be in labor. She was suffering from pain just as in the commencement of labor. A vaginal examination showed that there was a rounded tumor pushing downwards, the os uteri in the usual position, but not at all dilated or dilatable, and the cervix entirely absorbed or obliterated. I then did not doubt but what the enlargement was in the uterus, and that the condition was such as I have stated. Finding in a few days that there was no advance in labor, no attempt at dilatation of the os, I began to suspect that I might be wrong in my opinion. I asked Dr. Finnie to see her. We tried to introduce the uterine sound, but could not, so it was decided that the os had better be dilated and an exploration made. I could not enter the sound more than half an inch, but on trying to put in a laminaria tent, this latter took a course to the right side and went in easily to full extent. This was in the evening; next morning I put in a sponge tent to further dilate it. This went in the same way, and when dilated examined with my finger (under chloroform), but only could insert it about an inch and a half; thinking I could feel the membranes, it was a question whether an opening should be made in them or not. This I hesitated to do, as, if there was escape of contents, no uterine contraction might take place, so it was considered best to give ergot to induce them, and on their action to puncture the sac. This failed, however; its only effect was to again close the os more firmly. I again dilated with tents, being determined to explore more thoroughly and to puncture at the same time. On examination this time got my finger into the whole cavity of the uterus, which was directed to the right and shortened, and now found that there was nothing in it, the tumor apparently lying upon it and closely applied, as percussion on the abdomen could be plainly felt. Of course no attempt was made at puncturing through the uterus.

This condition was verified by Dr. Finnie, and we considered as she was now better than in September, and the tumor was getting smaller, to leave it alone and continue the supporting treatment.

The opinion I have formed from these examinations, the past history, etc., is this: That the impregnated ovum had been arrested in its downward descent to the uterus, in the tube close to the uterus on the left side. There grown, its distension gave rise to tne condition for which I was first called, probably rupture, that a new sac had grown around it, and in the entire growth had compressed the uterus and caused it to atrophy, and thus, as it occupied the median line, assumed the outline and position of the uterus. That there was a child I had no doubt, for I felt the fœtal movements. From a growth in such a cavity slight causes would induce its death, and not being in the uterine cavity no effort at labor would follow. The subsequent septic condition, the evidence of gas in the tumor, are what would follow if the child was dead, and possibly ulceration may have occurred into the intestinal canal, which would account for the fœtid condition of the discharge and the lessening size of the tumor which had been going on. I did not suspect it to be ovarian, until I made the examination in October, as it was not first observed at the side, but in the median line; besides, would it be possible for an ovarian disease to cause those changes to occur in the cervix which did occur, and caused it to be, as at present, entirely obliterated, and, at the same time, cause a total suppression of the menses for so many months.

The following are the notes of the case after the admission of the patient to the General Hospital, under Dr. Ross:

She was admitted on November 8, 1878. Patient is thin, pale, emaciated, with sunken eyes. She complains of great pain across the lower part of the abdomen, and frequent vomiting. The abdomen is smooth, prominent, and somewhat tense. The lower zone projects considerably more than the rest, but no definite tumor as from a gravid uterus can be seen. By pulsation the upper margin of this swelling is felt to be just above the level of the umbilicus. The whole region occupied by it is quite tender upon pressure, and throughout gives a hollow tympanitic note upon percussion. On the right side low down (iliac region) there can be felt a distinct fulness and hardness, and it is here that the tenderness is most marked and the greatest pain is felt. She is feverish (101° F.), quick, small, pulse, very fretful and irritable. Says she is very restless at night, and perspires a great deal. She was put upon a mixture of iron and quinine, and was ordered beef tea diet with port wine, and given doses of morphia at night to relieve pain.

Two days after admission she had a violent rigor, followed by high fever and profuse sweating, for which she got a hypodermic injection of morphia. Had two stools the last twenty-four hours—they were grayish-colored and very fœted, but contained no trace of fœtal debris.

On the 12th, pain, sweating, and weakness as before. No vomiting. No chills. This forenoon had two stools of similar characters to those last described, but containing in addition some small macerated fœtid bones without cartilages; these were three ribs, and a long bone, probably a tibia; also a number of pieces of tough, shreddy, greyish tissue, which are no doubt portions of decayed integuments. The next day she voided a well-formed temporal bone. Complaints of sharp cutting pain when her bowels are being moved. A digital examination of the rectum was made; it appeared natural throughout; there was an impression conveyed to the finger, just at the top of its utmost reach, as though this point were the lower border of a rounded opening, but no aperture could be felt. Two days subsequently, the general symptoms in the meantime remaing unaltered, the patient complained of very severe cutting pain in the rectum, so much so that it was feared a sharp portion of the fœtal skeleton might be impacted there, A second rectal exploration, however, proved that it was empty.

Nov. 16th.—A severe chill last night and another this morning, followed by a temperature of 105° F., great anxiety and oppression, and then profuse perspiration. Quinine, iron, beef tea, and wine, are being freely administered, with local anodyne applications and hypodermics of morphia at night.

This condition of irregular fever, with occasional rigors, alternating with drenching sweats, continued until the 27th instant, when new symptoms were developed. A teasing cough had lasted for two or three days, accompanied by a small amount of frothy expectoration, but physical examination showed nothing abnormal. On this day, however, she was suddenly attacked, about two P.M., with a violent stitching pain in the right lower ribs, with a most distressing squeezing sensation round the chest. Auscultation revealed a loud, rough and harsh pleuritic friction at lower part of the right latera region, extending less marked to the base of the lung behind. She was ordered morphia, the dose to be regulated at the discretion of the house-surgeon, and poultices. The next day the pain was relieved, but her pulse was very rapid, small, and compressible, and she gradually sank and died at two A.M. of the 30th instant.

*Post Mortem,* eight hours after death, (by Dr. Osler):—

Body that of a small, much emaciated woman; rigor mortis present; abdomen sunken; mammæ flattened and wasted; pamuculus adiposus very scanty.

On opening adomen, the parietal peritoneum is adherent from the naval downwards, and extending into the flanks. The attachments are separated without much difficulty, when a tumor is discovered, occupying the superior part of the pelvis, the organs of which are concealed by it. Above it extends nearly to the naval; the transverse colon is closely attached at the upper part and descends along the left side. On the right there are firmer adhesions with the lower coils of the ileum. The tumor is firmly fixed, occupies a central position, and is about the size of a child's head. On making a free incision the sac of an extra-uterine pregnancy is exposed, containing about ten ounces of dark, grayish-black material, looking like a mixture of coal ashes and water, and in this are disconnected bones of a fœtus, and discolored, and entirely devoid of soft parts. A peculiar and horribly fœtid odor is given off from the contents. The sac walls are about two millimetres in thickness at the front and lateral parts; thicker and more condensed in the pelvis. The lining membrane is roughened, of a dark, gray color, and in places quite black. On separating the adhesions of the sac to the pubis, the bladder and fundis uterus are exposed, when it is seen that the former (the sac) lies above and behind the uterus, extending between it and the rectum as low as the level of the os, but not much more to one side than the other, the balance, if any, being in favor of the right. A little to the left of the upper extremity of the sac is an oval orifice of communication with the sigmoid flexure of the colon, three-fourths inches in length, edges rounded and dark in color. On the right side there are several spots where perforation has almost taken place into the ileum, the coils of which could not be separated without tearing the sac-wall. In the broad ligament of the right side is a cyst the size of an apple, in communication with the main one by a narrow valvular opening, and filled with a similar ash-like material. It has thick walls, with a well-formed lining membrane. The fallopian tube terminates at the upper part of this cyst, being slightly dilated in its course and at the extremity. The ovary of this side could not be found, but whether accidentally cut away or destroyed in the growth of the sac cannot be positively said—probably the former. The ovary of left side not seen—probably left in the body—though it was thought that the entire contents of the pelvis had been removed. The fallopian tube of this side is cut off about one and a half inches from the uterus. The tissues of the broad ligaments on either side are much infiltrated and thickened, and on the right below the lesser sac there are several lines of suppuration passing down towards the vagina, and several of the veins contain thrombi The uterus is slightly enlarged, measuring five and a half inches in length, of which two and a half inches are made up by the cervix. Mucous membrane is soft, that of the cervix covered with a dirty semi-purulent secretion.

*Heart* presents nothing of note beyond five or six small perforations in the auricular septum.

*Pleura* over bases of both lungs inflamed and covered with flakes of lymph, about four ounces of exudation in the right side.

*Lungs.*—Posterior part of lower tube slightly collapsed and dark in color. One or two firmer spots are felt, which on section prove to be patches of pyæmic pneumonia, one of which is beginning to soften. In the lower tube of the left lung are several of these nodular, superficially placed spots; two have softened into small abscesses.

Nothing of note in the abdominal viscera.—*Canada Med-Record.*

